# Effects of ureteral stent removal using an extraction string following ureteroscopic lithotripsy: a systematic review and meta-analysis of randomized controlled trials

**DOI:** 10.3389/fsurg.2026.1718954

**Published:** 2026-02-19

**Authors:** Ce Dong, Hao Zheng, YuCheng Jiang, Weijie Chen

**Affiliations:** 1 Yuhuan People's Hospital, Taizhou, Zhejiang, China; 2 The Second People’s Hospital of Tongxiang, Jiaxing, Zhejiang, China

**Keywords:** lithotripsy, meta-analysis, systematic review, ureteral stent with a string, Visual Analog Scale

## Abstract

**Background:**

Ureteral stent removal using an extraction string after lithotripsy is gaining popularity; however, evidence regarding patient outcomes remains limited. This meta-analysis aimed to evaluate pain and complications associated between string-based and cystoscopic stent removal.

**Methods:**

A systematic search of PubMed, Web of Science, Embase, the Cochrane Library, and Scopus was conducted up to September 2025. Eligible randomized controlled trials (RCTs) compared string-based stent removal with cystoscopic stent removal. The primary outcome was pain, assessed using the Visual Analog Scale (VAS), with subgroup analyses performed by sex. Secondary outcomes included urinary tract infection (UTI) and other complications. Data synthesis was performed using Review Manager 5.4, and risk of bias and certainty of evidence were assessed using the GRADE approach.

**Results:**

Five RCTs involving 598 patients were included. Compared with cystoscopic stent removal, string-based stent removal significantly reduced pain [mean difference (MD) −2.49, 95% confidence interval (CI) −3.55 to −1.43, *p* < 0.01], particularly among women (MD −1.66, 95% CI −2.69 to −0.64, *p* < 0.01), while no significant pain reduction was observed among men (MD −1.05, 95% CI −3.75 to 1.64, *p* = 0.44). The incidence of UTI did not differ significantly between groups (risk ratio 1.45, 95% CI 0.48–4.42). Sensitivity analyses suggested instability of results, and stent migration could not be quantitatively assessed due to low event rates.

**Conclusion:**

Extraction string-based removal may be associated with lower pain, especially among female patients, without a clear increase in complications. However, the limited number of studies and substantial heterogeneity result in a low certainty of evidence, and further well-designed RCTs are needed to confirm these findings.

**Systematic Review Registration:**

https://www.crd.york.ac.uk/PROSPERO/view/CRD420251069187, PROSPERO CRD420251069187.

## 1 Introduction

Ureteral stents are widely used in urological surgeries, particularly following ureteroscopic lithotripsy, to maintain urinary drainage, prevent obstruction, and reduce the risk of edema and stricture formation (1). However, conventional stent removal requires cystoscopy, which was associated with morbidity, including pain, infection, and irritative voiding symptoms. These stent-related symptoms can have a substantial impact on patients’ daily activities and health-related quality of life. Ureteral stent insertion may also result in more serious complications, such as upward stent migration, sepsis, “forgotten stents,” or encrustation with stone formation, thereby increasing morbidity and healthcare costs (2).

The use of an extraction string for ureteral stent removal was first introduced by Siegel et al. as a simple technique to avoid general anesthesia and unnecessary urethral instrumentation in pediatric patients (3). In recent years, a growing body of literature has emerged on this topic, extending the application of extraction strings beyond pediatric patients to adult populations (4). Placement of a ureteral stent with an extraction string following ureteroscopy lithotripsy has been shown to reduce pain during stent removal without increasing the risk of associated complications, compared to conventional ureteral stents without an extraction string (4).

However, existing studies exhibit substantial variability in both the clinical application and terminology used to describe extraction strings (e.g., “tethered stents,” “stents with strings,” or “string stents”), which complicates direct comparisons across trials. Moreover, no systematic evaluation has been conducted to assess patient perspectives, including perceived benefits, discomfort, and overall acceptance of this technique. Existing narrative reviews and single-center comparative studies have yielded inconsistent findings and have not offered a comprehensive synthesis of randomized evidence. The primary aim of this systematic review and meta-analysis was to compare patient-reported Visual Analog Scale (VAS) pain scores at the time of ureteral stent removal between extraction-string-based and cystoscopic techniques. Secondary aims included evaluating postoperative complications, particularly urinary tract infections, and conducting prespecified subgroup analyses based on sex, stent dwell time, and the use of topical anesthesia.

## 2 Methods

### 2.1 Eligibility criteria

This study followed PRISMA guidelines as a checklist, and the study protocol was registered in the PROSPERO database (https://www.crd.york.ac.uk/PROSPERO/view/CRD420251069187). The inclusion criteria were defined according to the PICOS framework: the study population included patients who underwent ureteroscopy lithotripsy. The intervention group comprised patients whose ureteral stents were removed using an extraction string, while the control group underwent conventional stent removal via cystoscopy. The primary outcome was patient-reported pain during ureteral stent removal, measured using the VAS. Pain scores were analyzed for the overall patient population and stratified by sex to assess potential differences between male and female patients. The secondary outcome was the incidence of postoperative complications, particularly urinary tract infections (UTIs), as reported in the included studies. Only randomized controlled trials with accessible full-text publications were included, with no language restrictions. The exclusion criteria were as follows: (1) observational studies with control (cohort or case–control) or without control (cross-sectional studies or case series); (2) unpublished abstracts; and (3) non-primary research.

### 2.2 Search strategy

A systematic literature search was conducted across six databases (EMBASE, PubMed, the Cochrane Library, Web of Science, and Scopus). The data were updated through 14 September 2025. The following keywords were utilized in the search in PubMed: (((((ureteral stent [Title/Abstract]) OR (ureteric stent [Title/Abstract])) AND ((string [Title/Abstract]) OR (tether [Title/Abstract]))) AND ((removal [Title/Abstract]) OR (extraction [Title/Abstract]))) AND (randomized [Title/Abstract])) AND (stone [Title/Abstract]). There was no language limitation when searching. The search strategy and the respective databases are provided in the Supplementary Materials.

### 2.3 Study selection

Two researchers independently screened the titles and abstracts of identified studies. They evaluated the selected articles by full-text review and assessed their eligibility. Any disagreements were resolved by consulting with the third member of the review team.

### 2.4 Data extraction

Extracted study details included the first author, year of publication, sample size, study population, basic information [including age, body mass index (BMI), stone size, and sex], timing of pain assessment, stent type, stent dwell time, analgesia protocol at the time of stent removal, and reported outcomes. All data were retrieved by two researchers. Any discrepancies were resolved through discussion or, when necessary, consultation with the third member of the review team. When required details were not available in the published publications, we contacted the authors for additional information or clarifications.

### 2.5 Study risk of bias assessment

Two authors assessed the risk of bias of all studies using Review Manager 5.4 (5), evaluating domains such as random sequence generation, allocation concealment, blinding, incomplete outcome data, selective reporting, and other sources of bias. The assessment results were classified as having a high, low, or unclear risk of bias.

### 2.6 Data synthesis

Review Manager version 5.4 (Cochrane Collaboration) and StataMP version 14.0 were used for the meta-analysis. The inverse variance method was used to calculate the mean differences (MDs) and 95% confidence intervals (CIs) for continuous variables. A random-effects model was applied when heterogeneity was high; otherwise, a fixed-effects model was used. Risk ratios (RRs) were calculated for binary variables. Statistical heterogenicity was assessed using the *I*^2^ statistic. We categorized heterogeneity as low, moderate, or high when *I*^2^ < 25%, 26%–50%, or >50%, respectively (6). Results were considered statistically significant when the two-tailed *p*-value was ≤0.05. Baseline measurements were excluded to focus on treatment effects, in accordance with randomized controlled trial (RCT) design principles. Prespecified subgroup analyses were conducted based on stent dwell time (≤10 days vs. >10 days), timing of VAS assessment (immediate vs. non-immediate), use of topical anesthesia in the control group, and sex.

### 2.7 Assessment of publication bias

A formal assessment of publication bias was not performed because funnel plot analysis typically requires at least 10 studies to provide sufficient power for meaningful interpretation. With only five trials available, the reliability of such an assessment would be limited. However, the results may still be susceptible to publication bias due to the small number of available publications (5).

### 2.8 GRADE

The Grading of Recommendations Assessments, Development and Evaluation (GRADE) analysis was applied to the outcomes to evaluate the quality of evidence (7).

## 3 Results

### 3.1 Study selection

**Figure 1:**
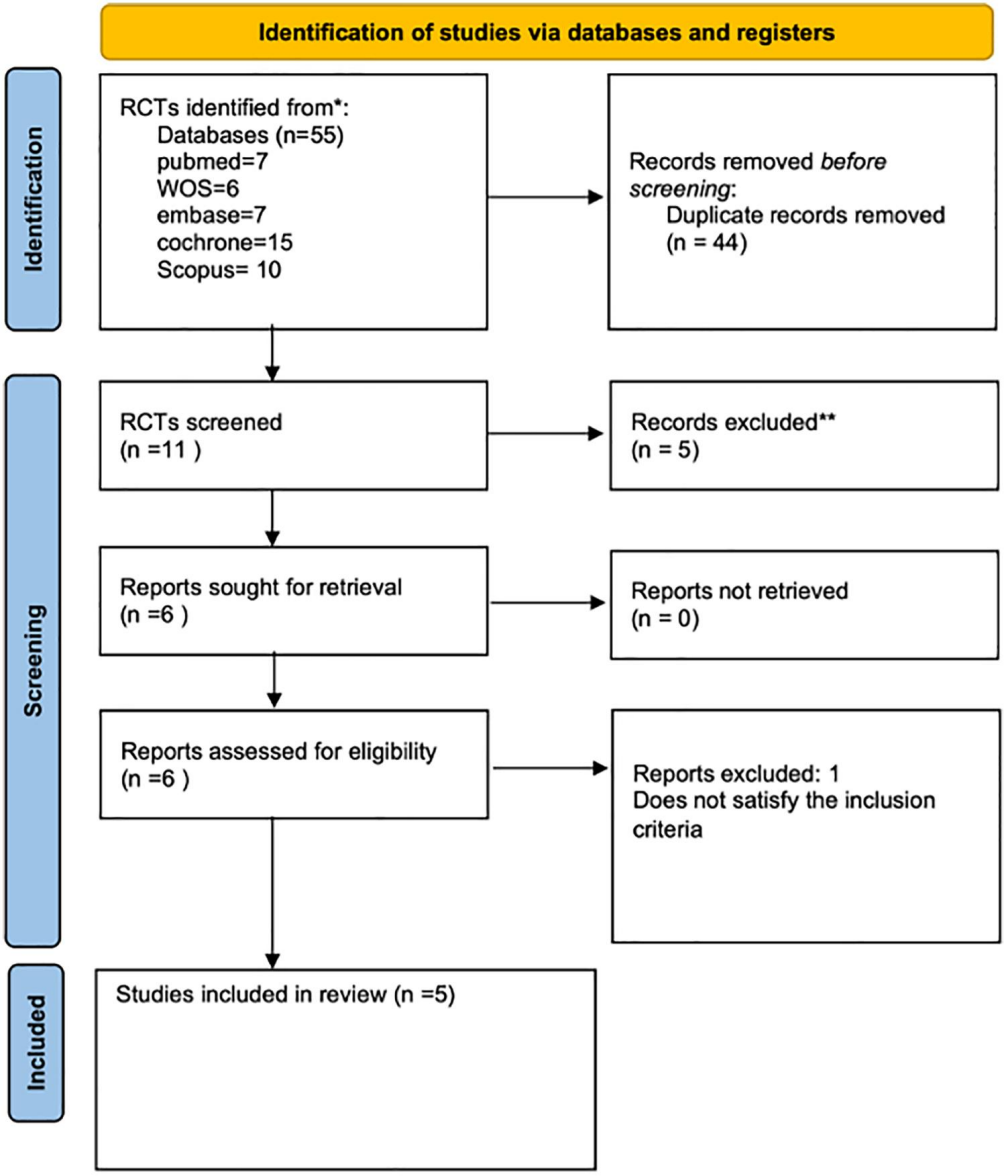
Preferred Reporting Items in Systematic Reviews and Meta-Analyses flowchart for study search. * Consider, if feasible to do so, reporting the number of records identified from each database or register searched (rather than the total number across all databases/registers). ** If automation tools were used, indicate how many records were excluded by a human and how many were excluded by automation tools (16).

A total of 55 articles were retrieved through the initial database search. Among them, 44 articles were removed as duplicates. Five studies were then excluded from the retrieved articles. Ultimately, five records were included in this systematic review. A detailed PRISMA flow diagram illustrating the study selection process is shown in Figure 1.

### 3.2 Characteristics of included studies

The five included studies originated from four different countries: China (8), the United States (4), the Republic of Korea (4), India (9), and Japan (10). The sample sizes ranged from a minimum of 68 participants (4) to a maximum of 149 participants (10). Three studies reported data stratified by sex (4, 10, 11). Regarding complications, three studies reported data on stent migration (4, 8, 10). However, two of these studies reported zero events, making it unsuitable to conduct a meta-analysis on these outcomes (4, 10). Two studies reported the incidence of UTIs (4, 10). For details, refer to Table 1. For missing data, we contacted the authors of the trial reports. The extracted data and characteristics of the included studies are summarized in Table 1. Since this study is a meta-analysis, the table presents only research findings included in at least two studies. For example, urinary irritation symptoms were not included because only one study (8) provided relevant data.

**Table 1:** Main characteristics of included studies evaluating the effect of stent removal via an extraction string.

Study ID	Year	Country	Timing of pain assessment	Stent type	Group	N	Stent dwell time (days)	Analgesia protocol at the time of stent removal	Stone size (mean ± SD, cm)	Age (mean ± SD, years)	Male (n)	BMI (mean ± SD, kg/m^2^)	Kidney Stone (n)	VAS total	VAS for men (*N*)	VAS for women (*N*)	Complication of UTI	Complication of Stent migration (n)	Randomization Method
Jiang	2025	China	Immediately	6F Double-J stent	Stent with a string	50	16.1 ± 4.5	Without analgesia	17.2 ± 7.5	50.69 ± 10.89	36	25.06 ± 3.278	25	0.86 ± 0.62	–	–	–	3	Unclear
					Cystoscope	50	60.5 ± 20.4	Local anesthesia	18.4 ± 11.2	48.77 ± 12.40	40	25.30 ± 2.171	29	5.23 ± 1.74	–	–	–	0	
Inoue	2019	Japan	Immediately	6F Double-J stent	Stent with a string	74	10	Without analgesia	11.5 ± 5.8	53.2 ± 12.9	50	28.2 ± 2.96	35	2.73 ± 2.37	2.86 ± 2.36 (50)	2.48 ± 2.44 (24)	4	0	Sealed-envelope method
					Cystoscope	75	10	Without analgesia	13.3 ± 6.8	55.5 ± 11.2	46	24.4 ± 4.5	33	5.67 ± 6.26	6.69 ± 6.18 (46)	4.02 ± 6.13 (29)	2	0	
Kim	2015	Republic of Korea	Immediately	6F Double-J stent	Stent with a string	58	5.97	Without analgesia	6.83 ± 2.04	50.97 ± 12.2	42	–	4	2.94 ± 1.35	3.19 ± 1.09 (42)	2.46 ± 1.71 (16)	–	–	random-number generator
					Cystoscope	56	6.28	Local anesthesia (lidocaine jelly)	7.64 ± 1.68	50.54 ± 14.28	36	–	7	4.23 ± 2.45	4.58 ± 2.23 (36)	3.54 ± 2.82 (20)	–	–	
Barnes	2014	USA	Immediately	6F Universa Soft	Stent with a strip	33	6.3	Without analgesia	12	48	12	32	14	2.5 ± 2.8	4.3 ± 3.8 (12)	2.1 ± 2.8 (21)	3	0	Sealed-envelope method
					Cystoscope	35	10.6	Local anesthesia (lidocaine jelly)	10	50	14	30.7	8	3.1 ± 2.8	2 ± 1.1 (14)	4.6 ± 2.9 (21)	3	0	
Agrawal	2014	India	Within 24 h	5F Double-J stent	Stent with a strip	83	10–14	Without analgesia	3.6	31	59	–	–	5.93 ± 0.5	–	–	–	–	Unclear
					Cystoscope	83	10–14	Tramadol +NSAIDs	3.8	33	62	–	–	3.081 ± 0.47	–	–	–	–	

BMI, body mass index; VAS, Visual Analog Scale; UTI, urinary tract infection; NSAIDs, non-steroidal anti-inflammatory drugs.

The included studies evaluated a variety of double-J (DJ) stents, including the Universa® Soft stent designed for short-term use with a self-removal string (4); the Percuflex Plus stent, which offers elasticity and shape memory for short- to midterm placement (11); a surgeon-modified tethered stent applied in tubeless percutaneous nephrolithotomy (9); the Inlay Optima stent with antiencrustation coating, typically used for approximately 10 days (10); and commercially available extraction string stents with a mean dwell time of approximately 16 days (8).

### 3.3 Risk of bias assessment

**Figure 2:**
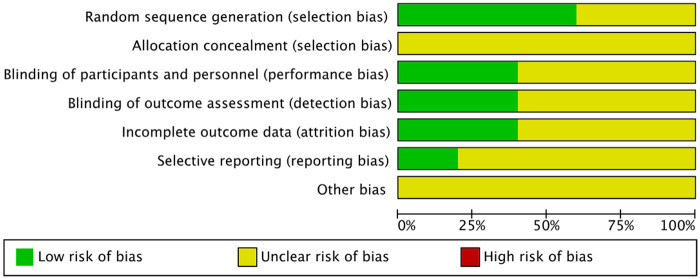
Graph for the risk of bias.

**Figure 3:**
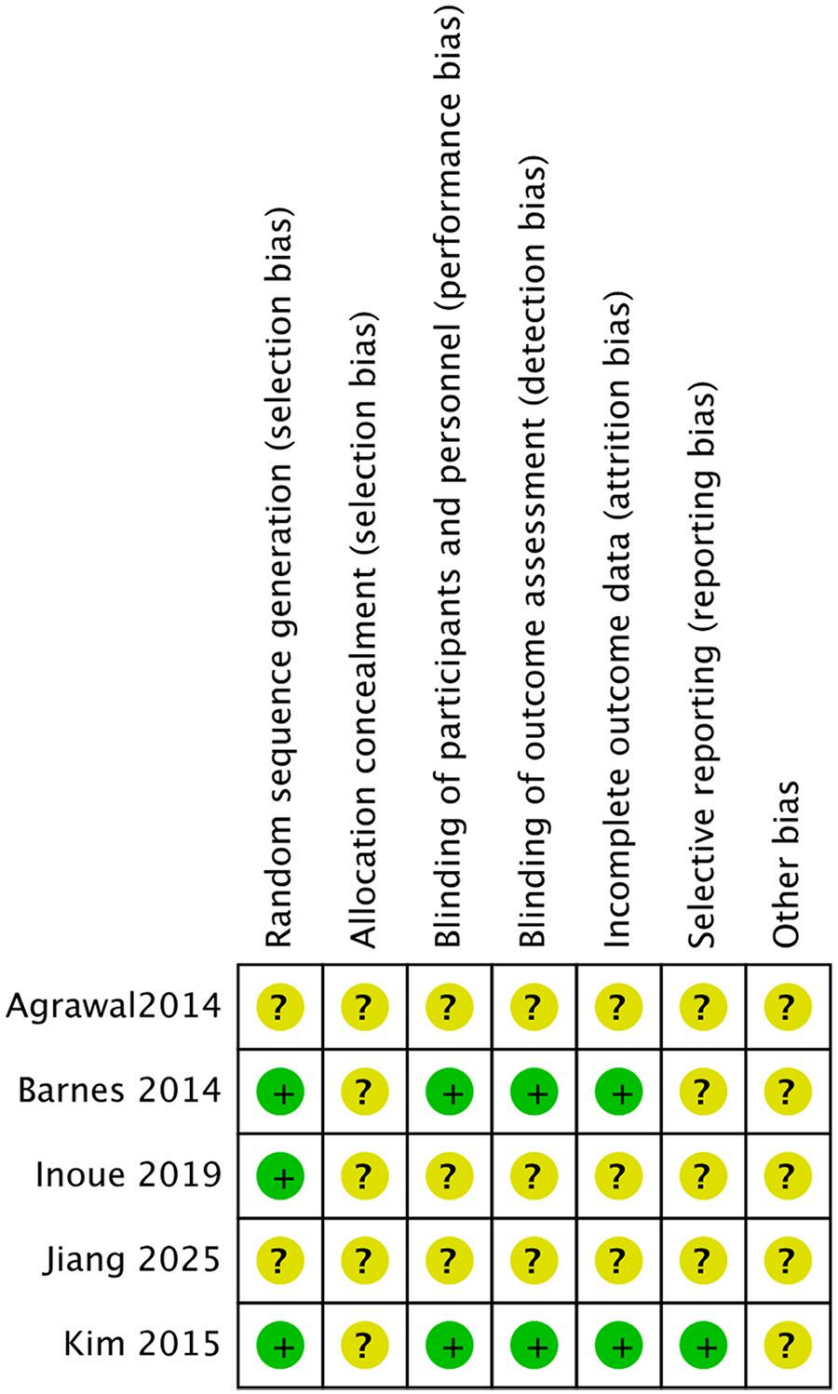
Summary of the risk of bias.

The visual representation of the risk of bias assessment is shown as a risk-of-bias graph (Figure 2) and a risk-of-bias table (Figure 3). The assessment showed a moderate risk of bias. All studies used randomization, but two did not describe the randomization methods in detail and were therefore rated as having an unclear risk of bias (8, 9). Blinding of participants and personnel was generally not feasible across all trials, as the interventions involved visible string extraction vs. cystoscopic removal; in addition, outcome assessment relied primarily on patient-reported measures, such as VAS pain scores and questionnaires. Therefore, these domains were judged as having an unclear risk of bias.

### 3.4 Primary outcomes

#### 3.4.1 VAS pain scores in the overall population

**Figure 4:**
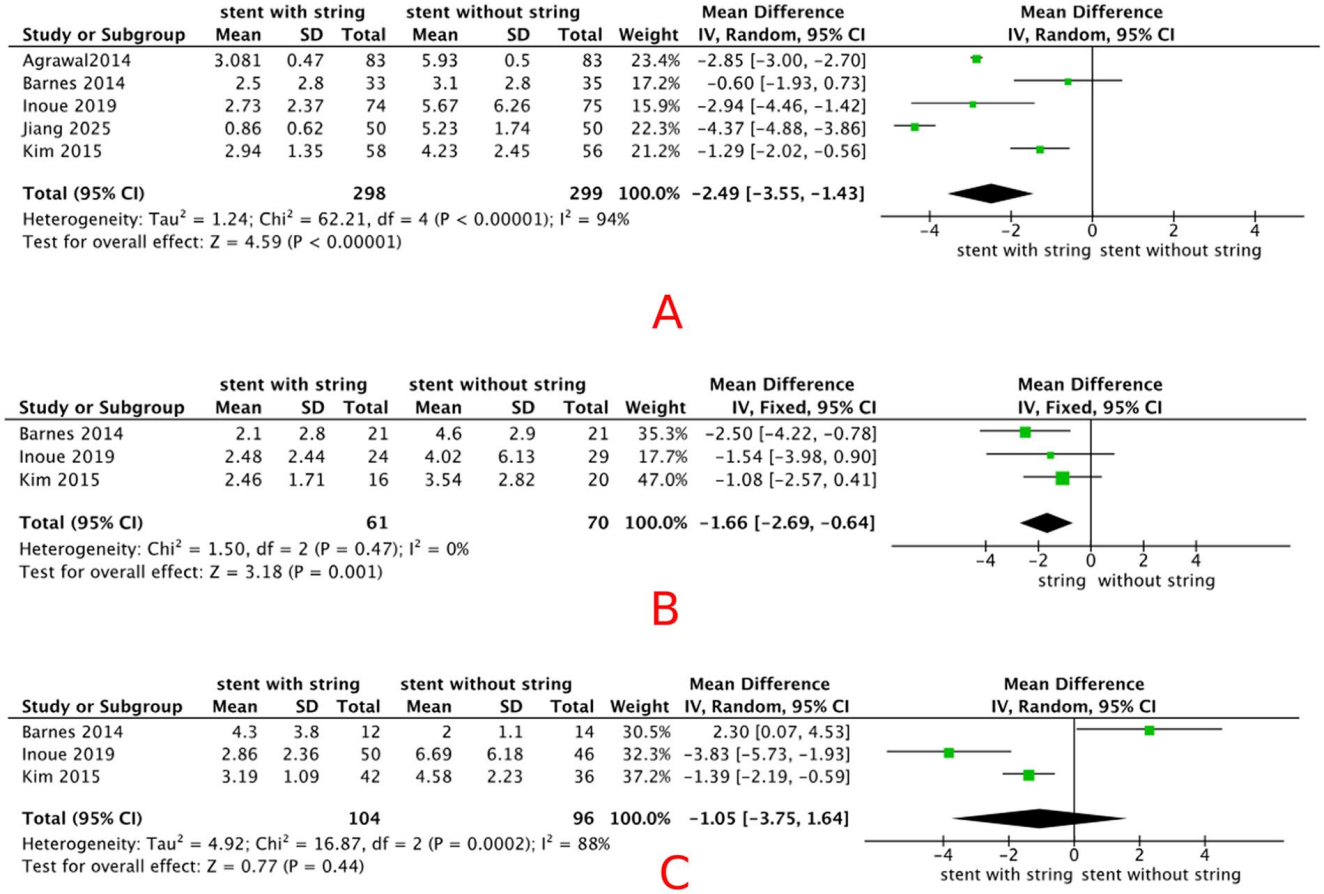
Forest plot of VAS pain scores during stent removal: **(A)** overall population, **(B)** female subgroup, and **(C)** male subgroup.

**Figure 5:**
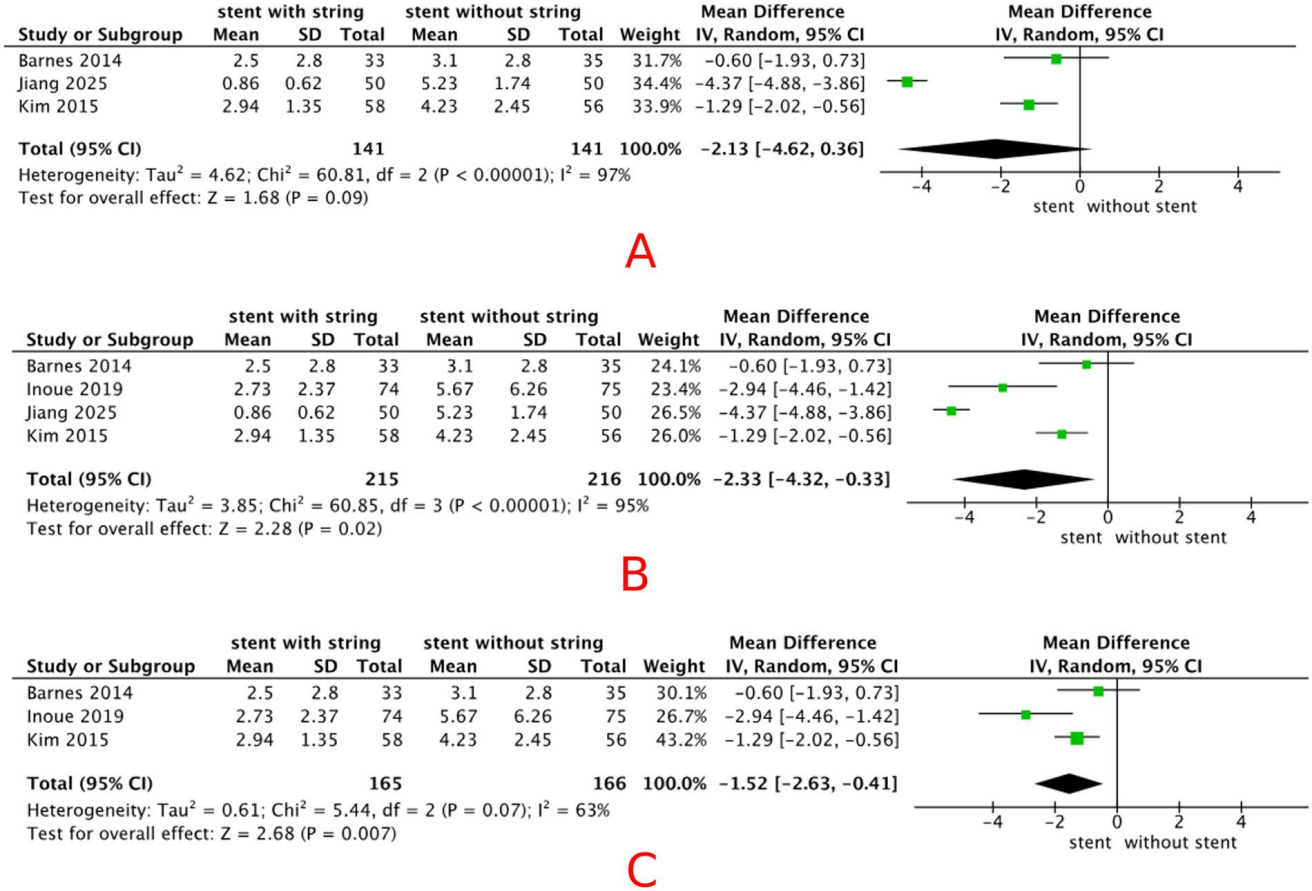
Subgroup analysis of the effect of stent removal via an extraction string. **(A)** control group received topical anesthesia, **(B)** VAS pain scores assessed immediately at the time of stent removal, and **(C)** stent dwell time ≤10 days.

**Figure 6:**
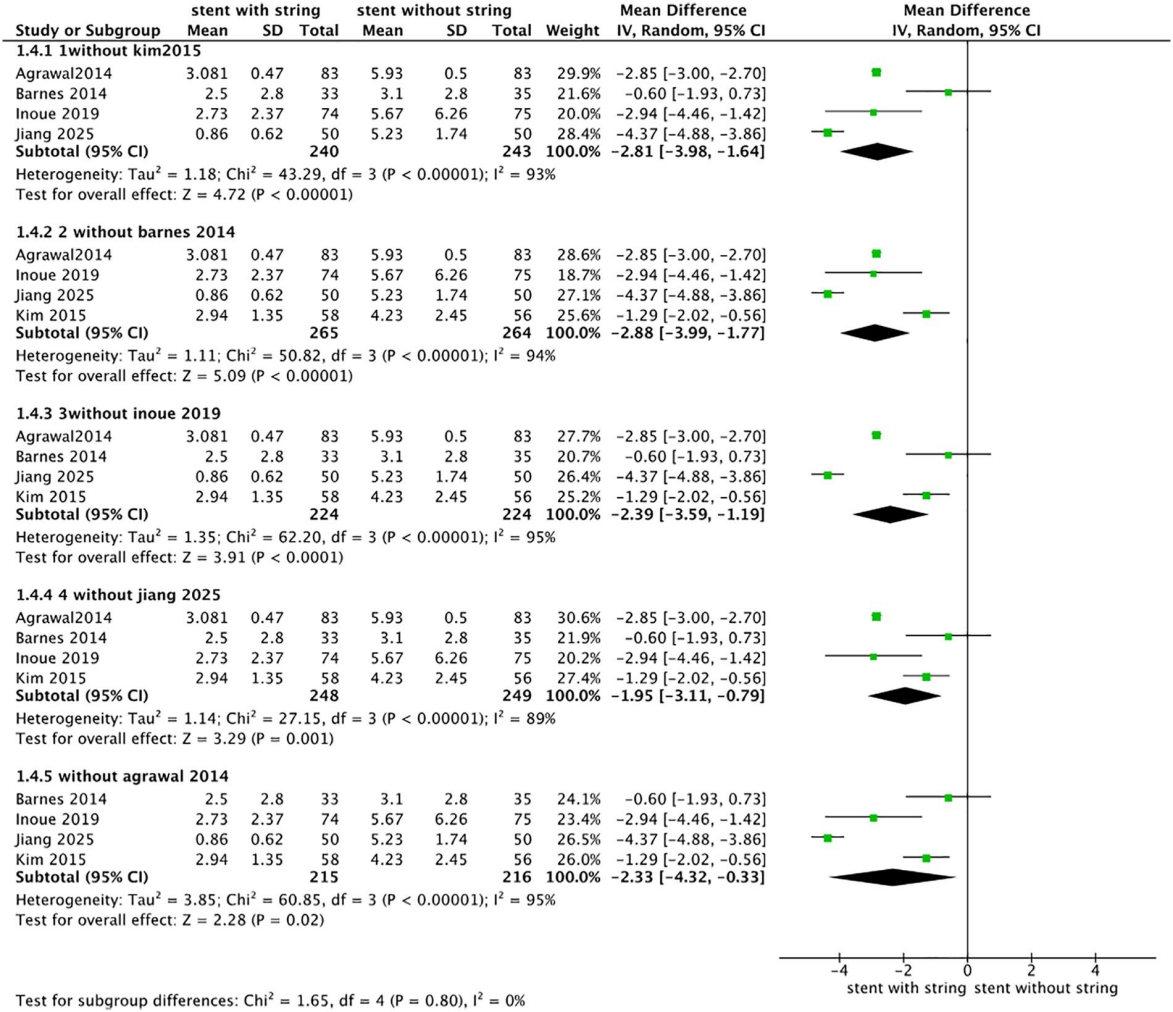
Sensitivity analysis of the effect of stent removal via an extraction string.

Five included studies (*n* = 598) reported VAS pain scores during stent removal. The pooled analysis showed that patients in the extraction string-based removal group reported significantly less pain than those in the cystoscopic removal group (MD = –2.49, 95% CI −3.55 to −1.43, *p* < 0.01), random-effects model), with a high degree of heterogeneity (*I*^2^ = 94%). Detailed results are shown in Figure 4. VAS pain scores were analyzed across three subgroups based on eligible studies, including (A) patients in the control group who received topical anesthesia, (B) studies in which VAS was assessed immediately at the time of stent removal, and (C) studies with a stent dwell time of ≤10 days. After subgrouping by stent dwell time ≤10 days, heterogeneity decreased to 63%. Detailed results are shown in Figure 5. Sensitivity analyses were therefore performed by sequentially excluding individual studies. When the study conducted in China (8) was excluded, the pooled effect size changed from −2.49 (95% CI −3.55 to −1.43) to −1.95 (95% CI: −3.11–−0.79), with the *I*^2^ value decreasing from 94% to 89%, the detail is shown in Figure 6. However, the overall direction of effect remained consistent across all sensitivity analyses; we were unable to identify a clear source of heterogeneity. The observed variability may be related to differences in stent types across groups, as summarized in Table 2. As indicated in the subsequent analysis, heterogeneity may also be partially attributable to sex-related differences. The main findings were robust despite the high heterogeneity.

**Table 2:** Comparison of stent types used in included RCTs.

Study + year	Stent type (brand/design)	Material/key features	Intended use
Barnes 2014	Universa Soft DJ stent (Cook Medical, 6F)	Softer polyurethane; short-term design	Short-term (≤14 days); string for self-removal at home
Kim 2015	Percuflex Plus DJ stent (Boston Scientific, 6F)	Polyurethane with elasticity and shape memory	Short- to midterm use
Agrawal 2014	Modified DJ stent (5F, 26 cm) with nylon tether	Generic polyurethane; surgeon-modified tether via nephrostomy	Tubeless PCNL
Inoue 2019	Inlay Optima DJ stent (Bard Medical, 6F)	Polyurethane with antiencrustation coating	Midterm use (≈10 days in trial)
Jiang 2025	Commercial string DJ stent (6F)	Marketed extraction-string stent	Short-term (≈16 days)

DJ, double-J stent; PCNL, percutaneous nephrolithotomy.

#### 3.4.2 VAS pain scores among female patients

Three included studies (*n* = 141) reported VAS pain scores during stent removal in female patients. The pooled analysis showed a significant decrease in pain with extraction string-based stent removal compared with cystoscopic stent removal [MD −1.66 (95% CI −2.69, −0.64), *p* < 0.01, fixed-effects model], with a low degree heterogeneity (*I*^2^ = 0%). Detailed results are shown in Figure 4.

#### 3.4.3 VAS pain scores among male patients

**Figure 7:**
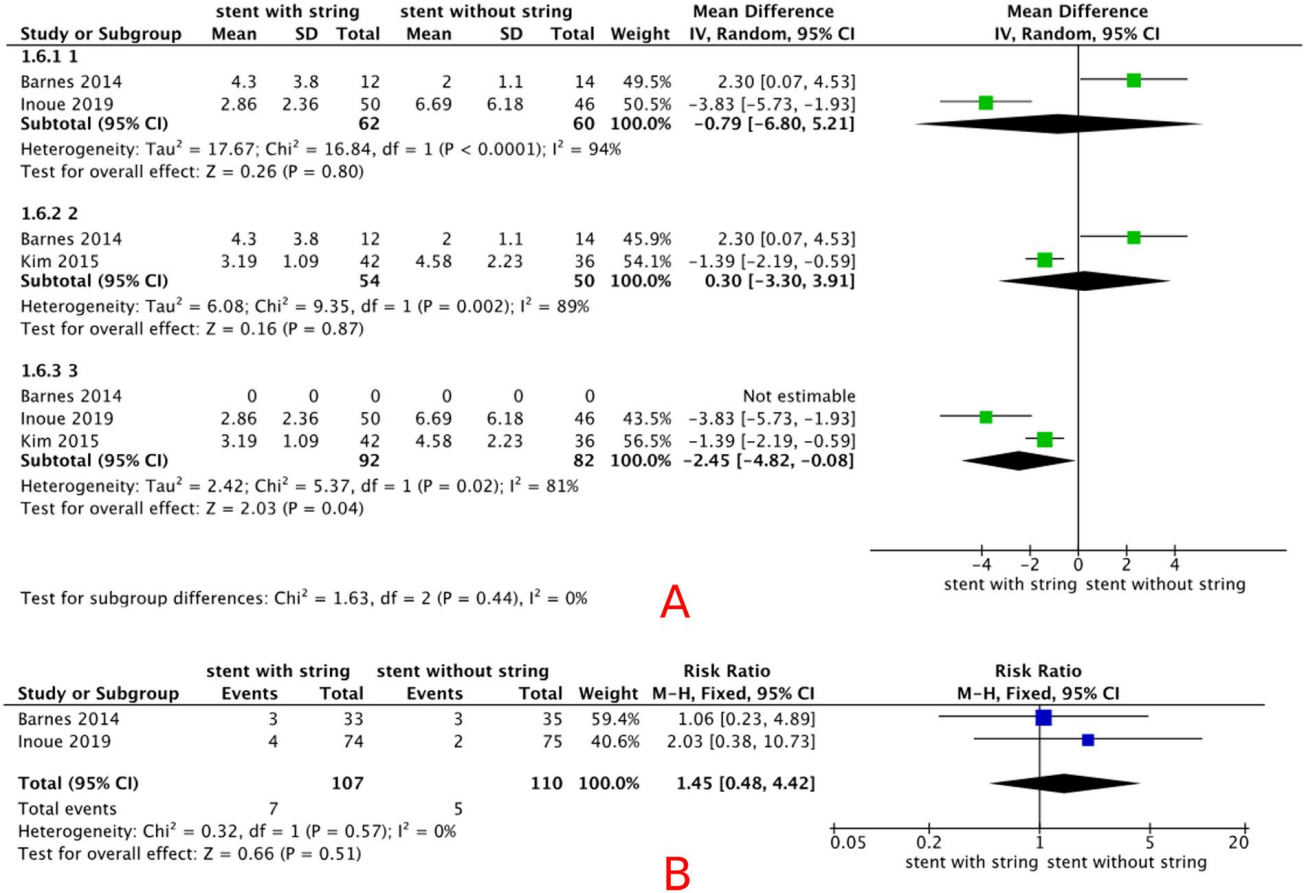
**(A)** sensitivity analysis of the effect of stent removal via an extraction string in male patients; **(B)** forest plot for the incidence of UTI.

Three included studies (*n* = 206) reported VAS pain scores during stent removal in male patients. The pooled analysis showed no statistically significant difference in pain between string-based and cystoscopic removal [MD −1.05 (95% CI −3.75, 1.64), *p* = 0.41, random-effect models], with a high degree heterogeneity (*I*^2^ = 88%). Detailed results are shown in Figure 4. The result was not robust in sensitivity analysis, and there was no reduction in heterogeneity. Detailed results are shown in Figure 7A.

### 3.5 Secondary outcomes

#### 3.5.1 Complication of stent migration

Although several studies reported outcomes related to stent migration, the number of events was zero in most studies, making meta-analysis infeasible.

#### 3.5.2 Complication of UTI

Two included studies (*n* = 217) reported the incidence of of UTI. The pooled analysis showed no statistically significant difference in UTI incidence between string-based and cystoscopic removal [RR 1.45 (95% CI 0.48, 4.42), *p* = 0.51, fixed-effects model], with a low degree of heterogeneity (*I*^2^ = 0%). Detailed results are shown in Figure 7B.

### 3.6 GRADE assessment of these studies

The GRADE assessment showed that the certainty of evidence ranged from moderate to very low for the reported results (Table 3). One outcome was downgraded due to inadequate randomization methods, one outcome was downgraded for imprecision because of wide confidence intervals, and two outcomes were downgraded due to high heterogeneity. In addition, all outcomes were downgraded for potential publication bias, as the limited number of included studies made it difficult to exclude its presence.

**Table 3:** GRADE assessment of outcomes.

Outcome	Study limitation	Imprecision	Inconsistency	Indirectness	Publication bias	GRADE
VAS pain scores in the overall population	Downgraded due to unclear randomization methods in some studies.	No downgrade	downgrade due to I2 = 94%	No downgrade	Downgraded due to the limited number of included studies.	Very low
VAS pain scores among female patients	No downgrade	No downgrade	No downgrade	No downgrade	Downgraded due to the limited number of included studies.	Moderate
VAS pain scores among male patients	No downgrade	Downgraded due to wide confidence intervals.	Downgrade due to I2 = 88%	No downgrade	Downgraded due to the limited number of included studies.	Very low
Complication	No downgrade	No downgrade	No downgrade	No downgrade	Downgraded due to the limited number of included studies.	Moderate

VAS, Visual Analog Scale.

## 4 Discussion

In this meta-analysis, we compared the removal of ureteral stents using an extraction string with conventional cystoscopic removal. The analysis suggests that stent removal via an extraction string may be associated with reduced pain in the overall population, particularly among female patients, although high heterogeneity as observed across studies (*I*^2^ = 94%). Subgrouping by stent dwell time ≤10 days reduced heterogeneity to 63%, suggesting that dwell time may contribute to the observed variability. Nonetheless, as heterogeneity persisted at a moderate level, this observation should be interpreted with caution, and additional factors, such as stent type and patient demographics, may also play a role. However, no significant pain reduction was observed in male patients. These findings were not robust in sensitivity analyses. Regarding UTI, no significant difference was found between the two stent removal methods. In the overall analysis, string-based removal was associated with a mean reduction of 2.49 points on the VAS compared with cystoscopic removal. From a clinical perspective, a difference of this magnitude exceeds the commonly cited minimal clinically important difference of approximately 1.0–1.5 points for acute pain, suggesting that the observed pain reduction is likely to be meaningful for many patients.

The very high heterogeneity observed for the primary outcome (*I*^2^ = 94%) substantially limits the certainty of the pooled estimate and suggests that the true effect may vary across settings. Although subgroup analyses by stent dwell time (≤10 days) reduced the *I*^2^ to 63%, heterogeneity remained moderate. This finding indicates that data pooling is informative for exploring overall trends, but the summary effect should be interpreted with caution rather than as a precise estimate. Potential contributors to heterogeneity include differences in stent type, analgesia protocols at the time of stent removal, timing of VAS assessment, surgical indication and technique, and patient-specific factors such as sex and urethral anatomy. The diversity of stent designs and materials summarized in Table 2 may also have contributed to between-study differences in pain and complication outcomes. Although several subgroup analyses were performed, they were not exploratory *post-hoc* analyses. All subgroup variables (sex, stent dwell time, timing of VAS assessment, use of topical anesthesia) were prespecified in the PROSPERO protocol, as previous urological and pain-assessment literature suggests that these factors may influence perceived pain during ureteral instrumentation. Although most studies assessed VAS immediately at the time of stent removal, one study assessed VAS after a short delay; therefore, subgrouping allowed us to evaluate whether assessment timing contributed to heterogeneity.

The observed sex-based differences and study heterogeneity may be attributed to anatomical and methodological factors. The longer and more curved male urethra may reduce the comfort and effectiveness of string-based removal, whereas the shorter female urethra allows easier manipulation and more consistent pain relief. Additional variability may arise from differences in study design, stent dwell time, stent type, analgesic use, timing of pain assessment, and patient preparation. These factors likely contributed to inconsistent findings and a lack of robustness in the sensitivity analyses. High heterogeneity in pain-related outcomes has also been reported in other meta-analyses of analgesic interventions, underscoring the well-recognized variability inherent in pain assessment research (12). Similar methodological challenges related to outcome heterogeneity and variability in subjective or observer-dependent assessments have been described in meta-analyses from other clinical fields, where differences in assessment timing, scoring tools, and study design substantially contributed to between-study heterogeneity (13). Standardized protocols and stratified reporting are needed in future research to improve comparability and clinical applicability. These methodological observations support our interpretation that the heterogeneity in VAS outcomes across trials is likely multifactorial rather than attributable to a single parameter, such as dwell time, further emphasizing the need for standardized pain-assessment protocols in future studies.

In addition to the pooled analgesic effects, several practical considerations may influence when extraction string-based removal is clinically appropriate. Randomized data have shown that string-based removal is particularly advantageous in short-term stenting (≤7–10 days), where the risk of discomfort from the extraction string is lower and outpatient removal is typically preferred (4). Accidental early dislodgement of the stent, although uncommon, has been reported in 1%–2% of patients and may lead to unplanned cystoscopy or premature stent removal (11). Importantly, extraction strings enable self-removal of ureteral stents at home, a feature that many patients find convenient and less disruptive than returning for cystoscopic removal. However, some patients report anxiety when manipulating the string or concern about accidental dislodgement of the stent (14). From a health-system perspective, eliminating cystoscopy can reduce clinic resource utilization and procedural costs without increasing the risk of UTI, making string-based removal a potentially cost-effective option when patient preference aligns with clinical appropriateness (15). Collectively, these factors emphasize that decisions regarding string-based vs. cystoscopic removal should incorporate not only pain outcomes but also patient preferences, stent dwell time, perceived comfort, and logistical and economic considerations.

According to the GRADE assessment, the certainty of evidence ranged from very low to moderate, mainly due to unclear randomization methods, wide confidence intervals, high heterogeneity, and the small number of available trials; consequently, our conclusions should be regarded as cautious and hypothesis-generating rather than definitive.

We also acknowledge several limitations in our study. First, the included studies did not provide detailed descriptions or standardized criteria for the timing of VAS assessment. Second, based on the Cochrane risk of bias assessment, while most of the included studies described the random allocation method, some did not provide details on allocation concealment or personnel blinding. This made it difficult to determine whether proper blinding of participants and personnel was implemented. Third, the included studies were limited to short observation periods and lacked long-term follow-up. The methodological quality of clinical trials needs further improvement. Fourth, the study may be susceptible to publication bias due to the limited number of available publications. In addition, the total sample size was modest, and some subgroup analyses—particularly those restricted to male patients or UTI outcomes—were underpowered; therefore, non-significant findings in these subgroups may reflect limited statistical power rather than a true absence of effect. Therefore, well-designed, multicenter randomized trials with larger sample sizes are needed to provide more robust evidence on the comparative effectiveness and safety of string-based vs. cystoscopic stent removal.

## 5 Conclusion

This meta-analysis suggests that ureteral stent removal via an extraction string may reduce pain compared with cystoscopic removal, particularly among female patients, although the certainty of this effect remains limited. No clear advantage was demonstrated in male patients, and sensitivity analyses indicated limited robustness of the pooled estimates. The incidence of UTI appeared to be similar between the two methods. Given the overall very low to moderate certainty of evidence, largely due to high heterogeneity and small sample sizes, these findings should be interpreted with caution. Extraction string-based removal may be considered as an alternative in selected patients, particularly females; however, further high-quality trials are needed to confirm these results and to inform patient-centered guidelines.

## Data Availability

The original contributions presented in the study are included in the article/Supplementary Material, further inquiries can be directed to the corresponding author.
