# Supplementary material for: Effects of ureteral stent removal using an extraction string following ureteroscopic lithotripsy: a systematic review and meta-analysis of randomized controlled trials
Source: Front Surg. 2026 Feb 19;13:1718954. doi: 10.3389/fsurg.2026.1718954 (PMC12960589; doi:10.3389/fsurg.2026.1718954)
Supplement: Supplementary file 1 [file Supplementaryfile1.docx]

search from inception to september 14, 2025

pubmed:

(((((ureteral stent[Title/Abstract]) OR (ureteric stent[Title/Abstract])) AND ((string[Title/Abstract]) OR (tether[Title/Abstract]))) AND ((removal[Title/Abstract]) OR (extraction[Title/Abstract]))) AND (randomized)) AND (stone)

Scopus

Search strategy: TITLE-ABS-KEY ( randomized ) AND ( "ureteral stent" OR "ureteric stent" ) AND ( "string" OR "tether" ) AND ( "removal" OR "extraction" ) AND ( "stone" )

Embase:

Search strategy: ('ureteral stent':ti,ab,kw OR 'ureteric stent':ti,ab,kw)

AND

(randomized:ti,ab,kw)

AND

('string':ti,ab,kw OR 'tether':ti,ab,kw)

AND

('removal':ti,ab,kw OR 'extraction':ti,ab,kw)

AND

('stone':ti,ab,kw)

Cochrone

Search strategy:

#1 ("ureteral stent"):ti,ab,kw

OR ("ureteric stent"):ti,ab,kw

#2 ("string"):ti,ab,kw

OR ("tether"):ti,ab,kw

#3 ("removal"):ti,ab,kw

OR ("extraction"):ti,ab,kw

#4 ("stone"):ti,ab,kw

#5 #1 AND #2 AND #3 AND #4

Web of Science

Search strategy:

TS=(

"ureteral stent"

OR "ureteric stent"

)

AND

TS=(

removal

OR extraction

)

AND

TS=(

stone

)

AND

TS=(

random*

OR trial

)
